# Supplementary material for: Major Histocompatibility Complex Class I Chain-Related α (MICA) STR Polymorphisms in COVID-19 Patients
Source: Int J Mol Sci. 2022 Jun 23;23(13):6979. doi: 10.3390/ijms23136979 (PMC9266713; doi:10.3390/ijms23136979)
Supplement: Supplementary file 1 [file ijms-23-06979-s001.zip › Supplementary Table S1.pdf]

Supplementary Table S1. Linkage disequilibrium analysis between major histocompatibility complex class I chain-related gene A (MICA) and human leucocyte antigen (HLA)-B in COVID-19 patients.

	**FHO**	**FHE**	**D′**	**r^2**	**χ^2**	**p-value**
*HLA-B*07/MICA*A51*	83	18.91	0.9846	0.3251	307.5664	<0.0001
*HLA-B*41/MICA*A6*	15	5.22	1	0.0302	28.5839	<0.0001
*HLA-B*14/MICA*A5*	11	6.66	0.0814	0.0036	3.3975	0.065
*HLA-B*35/MICA*A9*	51	17.45	0.4271	0.0927	87.6963	<0.0001
*HLA-B*50/MICA*A6*	25	8.69	1	0.0509	48.172	<0.0001
*HLA-B*40/MICA*A5*	24	4.11	0.6048	0.1192	112.8063	<0.0001
*HLA-B*27/MICA*A4*	25	3.36	1	0.1750	165.5967	<0.0001
*HLA-B*18/MICA*A4*	75	10.07	1	0.5553	525.3085	<0.0001
*HLA-B*57/MICA*A9*	34	6.18	1	0.1678	158.7039	<0.0001
*HLA-B*44/MICA*A51*	43	35.8	0.0584	0.0024	2.2462	0.1339
*HLA-B*51/MICA*A6*	58	23.3	0.7940	0.0901	85.2621	<0.0001
*HLA-B*15/MICA*A5*	30	4.33	0.7404	0.1888	178.6118	<0.0001
*HLA-B*44/MICA*A6*	114	55.3	0.5661	0.1214	114.8525	<0.0001
*HLA-B*35/MICA*A6*	13	33.39	-0.6106	0.0225	21.2424	<0.0001
*HLA-B*40/MICA*A51*	12	8.33	0.1280	0.0023	2.1705	0.1407
*HLA-B*08/MICA*A51*	42	9.46	1	0.1599	151.2503	<0.0001
*HLA-B*14/MICA*A6*	49	20.87	0.7189	0.6565	62.0934	<0.0001
*HLA-B*39/MICA*A9*	16	3.09	0.9281	0.0709	67.1013	<0.0001
*HLA-B*38/MICA*A9*	33	6.36	0.9302	0.1496	141.5036	<0.0001
*HLA-B*53/MICA*A9*	19	3.64	0.9389	0.0857	81.0496	<0.0001
*HLA-B*55/MICA*A4*	16	2.15	1	0.1109	104.9563	<0.0001
*HLA-B*13/MICA*A51*	13	2.93	1	0.0479	45.3604	<0.0001
*HLA-B*49/MICA*A6*	28	9.74	1	0.0572	54.1123	<0.0001
*HLA-B*52/MICA*A6*	16	5.91	0.9098	0.0284	26.8729	<0.0001
*HLA-B*35/MICA*A5*	32	10.66	0.2501	0.0566	53.5272	<0.0001
*HLA-B*58/MICA*A9*	10	1.82	1	0.0481	45.4808	<0.0001

Only the haplotypes with frequency higher than 1% are shown. The false discovery rate (FDR)-based Benjamini and Lui (BL) method was used to the correction for multiple testing. P-value was considered significant only when it was smaller than its corresponding BL critical value. HFO: haplotypic frequency observed; HFE: haplotypic frequency expected; D′: 5 standardized D; r^2: correlation index.
