# Supplementary material for: Major Histocompatibility Complex Class I Chain-Related α (MICA) STR Polymorphisms in COVID-19 Patients
Source: Int J Mol Sci. 2022 Jun 23;23(13):6979. doi: 10.3390/ijms23136979 (PMC9266713; doi:10.3390/ijms23136979)
Supplement: Supplementary file 1 [file ijms-23-06979-s001.zip › Supplementary Table S2.pdf]

Supplementary Table S2. HLA-B allele frequencies.

	Controls		Asymptomatic		Moderate Patients		Severe Patients		COVID-19 Patients	
	**Frequency**	**Percentage**	**Frequency**	**Percentage**	**Frequency**	**Percentage**	**Frequency**	**Percentage**	**Frequency**	**Percentage**
**HLA-B*07**	121	9.8	9	13.64	60	9.04	17	8.21	86	9.20
**HLA-B*08**	61	4.9	3	4.55	36	5.42	4	1.93	43	4.60
**HLA-B*13**	26	2.1	1	10.52	9	1.36	3	1.45	13	1.39
**HLA-B*14**	67	5.4	4	6.06	45	6.78	13	6.28	62	6.63
**HLA-B*15**	62	5	4	6.06	27	4.07	9	4.35	40	4.28
**HLA-B*18**	130	10.5	5	7.58	58	8.73	14	6.76	77	8.24
**HLA-B*27**	43	3.5	3	4.55	14	2.11	9	4.35	26	2.78
**HLA-B*35**	125	10.1	4	6.06	74	11.14	21	10.14	99	10.59
**HLA-B*37**	13	1.1	0	0	0	0	0	0	0	0
**HLA-B*38**	31	2.5	2	3.03	23	3.46	10	4.83	35	3.74
**HLA-B*39**	17	1.4	3	4.55	13	1.96	2	0.97	18	1.93
**HLA-B*40**	37	3	4	6.06	28	4.22	5	2.42	37	3.96
**HLA-B*41**	15	1.2	2	3.03	10	1.51	3	1.45	15	1.60
**HLA-B*44**	191	15.5	12	18.18	110	16.57	39	18.84	161	17.22
**HLA-B*45**	20	1.6	0	0	13	1.96	0	0	13	1.39
**HLA-B*47**	3	0.2	0	0	0	0	0	0	0	0
**HLA-B*48**	1	0.1	0	0	0	0	0	0	0	0
**HLA-B*49**	31	2.5	1	1.52	20	3.01	7	3.38	28	2.99
**HLA-B*50**	39	3.2	1	1.52	14	2.11	10	4.83	25	2.67
**HLA-B*51**	113	9.2	3	4.55	48	7.23	18	8.70	69	7.38
**HLA-B*52**	19	1.5	1	1.52	10	1.51	6	2.90	17	1.82
**HLA-B*53**	15	1.2	2	3.03	15	2.26	3	1.45	20	2.14
**HLA-B*55**	9	0.7	1	1.52	13	1.96	2	0.97	16	1.71
**HLA-B*56**	3	0.2	0	0	0	0	0	0	0	0
**HLA-B*57**	26	2.1	1	1.52	22	3.31	12	5.80	35	3.74
**HLA-B*58**	16	1.3	0	0	0	0	0	0	0	0

*HLA alleles with frequency under 1% have been removed. No significant differences were found for the possible comparations.
